# Clinical and histopathological features of breast tumors in women: a cross-sectional study at three hospitals in the Kingdom of Saudi Arabia

**DOI:** 10.11604/pamj.2021.39.267.30341

**Published:** 2021-08-24

**Authors:** Mohamed Abdel Razik, Alhumaidi Mazyad Alsubaie, Hassan Mohammed Alsetri, Khalid Abdulrahman Albassam, Abdulrahman Omar Alkhurayyif, Mazen Mohammed Altamimi, Sultan Mohammed Alanazi

**Affiliations:** 1 General Surgery Department, College of Medicine, Prince Sattam Bin Abdulaziz University, Al-Kharj, Saudi Arabia,; 2 College of Medicine, Prince Sattam Bin Abdulaziz University, Al-Kharj, Saudi Arabia

**Keywords:** Breast cancer, tumors, fibroadenoma, ductal carcinoma, lobular carcinoma, Saudi Arabia

## Abstract

**Introduction:**

there is a dearth of data on the epidemiology of breast tumors in the Kingdom of Saudi Arabia (KSA). This study aimed to determine the demographics, clinical patterns, and their association with histopathological types of breast tumors among females presently residing in KSA.

**Methods:**

a multi-centric, cross-sectional study including female patients with symptoms suggestive of breast tumor was conducted at three hospitals in KSA from February 2019 to February 2020. The patient´s electronic health records were used to collect information related to their demographics, clinical findings including comordbities and symptoms and investigations. Binary logistic regression models were used to determine factors associated with the breast tumors.

**Results:**

a total of 270 female patients were included in the study. The most frequently encountered symptom was a breast lump (95.9%, n = 259), followed by pain (18.9%, n = 51). More than half the population (53%, n = 143) had a histopathological diagnosis of fibroadenoma. Multivariate analysis revealed that patients > 46 years of age were less likely to present with fibroadenoma (AOR: 0.049 95% CI 0.02 - 0.15; p < 0.005). Those in the 31 - 45 years age group were less likely to present with ductal/lobular/papillary carcinomacompared to the less than 30 years group (AOR: 0.42, 95% CI 0.18 - 0.97; p = 0.04).

**Conclusion:**

in this population of Saudi women with symptoms suggestive of breast tumor, those aged less than 40 years were more likely to have a fibroadenoma whereas those above 40 years were more likely to have breast cancer.

## Introduction

Breast cancer is one of the most common and leading cause of mortality among women worldwide. Globally, new cases of women breast cancer were reported to be 2.1 million in the year 2018 [1]. Furthermore, breast cancer incidence is increasing in all age groups [2] primarily in those countries with lower social development index (SDI) [3]. Breast cancer is frequently reported in Saudi women with a prevalence of 21.8% [4]. According to World Health Organization (WHO), about 3954 people were diagnosed with breast cancer and 1095 people died of it in Kingdom of Saudi Arabia (KSA) in the year 2020 [5]. In this context, breast cancer has been reported to be 9th leading cause of mortality in KSA [4]. Like the other countries of the world, breast cancer incidence is expected to increase in next few decades [4]. Breast cancer accounts for 29% of all cancers reported in KSA [6].

Clinically, breast cancer usually presents as a lump in breast, pain, change in breast size, or discharge from nipple [7]. The most frequent presentation is a breast swelling [8]. Factors like age, lifestyle and family history play important role in the development of breast cancer [9]. Breast tumors may be classified into various subtypes based on specifichistology, anatomical origin, or hormone receptivity. The most frequent type of breast cancer among Saudi women has been reported to be ductal carcinoma (81.80%) followed by lobular carcinoma (3.40%) [10]. Different subtypes of breast cancer possess different characteristics and prognoses. Ductal carcinoma in situ (DCIS) refers to noninvasive and non-infiltrating neoplasms which remain localized within mammary ducts [11]. Likewise, lobular carcinoma in situ (LCIS) refers to those from the lobules [12]. DCIS and LCIS are associated with higher risk of invasive breast cancer [13]. Invasive ductal carcinoma that grows like a cohesive mass often presents as a discrete palpable lump. On the contrast, the invasive lobular carcinoma consisting of non-cohesive cells organized in a linear pattern are not well palpable and often present late with multifocal, multicentric, or contralateral involvement [14]. Therefore, each type of breast cancer presents differently which may require different treatment modalities and prognosis [15].

Although many articles on breast cancer have been published from KSA [16, 17]; however, detailed information on the histopathological types of breast cancer and their relationship with clinical presentation is still lacking. Therefore, the objectives of the study wereto determine the demographics, clinical presentations, and their association with types of breast tumors among females presently residing in KSA.

## Methods

### Study design and setting

This was a multi-centric, cross-sectional study conducted at three hospitals in the Kingdom of KSA viz. King Khalid Hospital and Prince Sultan Center for Health Services, Prince Sattam Bin Abdulaziz University Hospital in Al Kharj, KSA, and Al Kharj Military Industries Corporation hospital. The study period was from February 2019 to February 2020.

### Study population

We included 270 cases in this study. Based on the prevalence of histopathologically diagnosed *Lobular Carcinoma* in a large retrospective medical record database study, 344 (7.8%), the minimum sample size required was calculated to be 226, for a 95% confidence interval with a 2.5% margin of error [18]. The criteria for inclusion in this study were females, aged 17 and above, presently residing in the KSA, who presented to the general surgery clinics, surgical wards, and emergency surgery clinics at the three hospitals during the study period, with symptoms suggestive of breast tumors such as breast lump or change in breast size, mastodynia, breast abscess, nipple discharge. Male patients, those in the pediatric age group, those with incomplete or missing data points, those who refused diagnostic testing, patients for whom we could not get permission from their legal guardians, and those who did not consent to participate in the study were excluded.

### Data collection

The patient´s electronic health records were used to collect information related to their age, ethnicity, clinical history of present illness, including any symptoms suggestive of breast tumors, family history of breast cancer, and presence of comorbid diabetes mellitus. We also assessed their medical reports related to diagnostic investigations such as breast mammogram, histopathological diagnosis with fine needle aspiration cytology (FNAC) or biopsy of breast lump and lymph node biopsy. Further, we collected information regarding the type of surgeries performed for breast tumor treatment.

### Statistical analysis

The data was analyzed using IBM SPSS Statistics version 22.0 for Windows (Chicago, Illinois, USA). A descriptive analysis was performed to characterize the study population demographics, medical history, diagnostic investigations, histopathological diagnosis and surgical interventions. Categorical variables are expressed as n (%) while age, the only continuous variable is expressed as mean ± standard deviation. We constructed binary logistic regression models to determine factors associated with the four most frequently occurring histopathological diagnoses in our study, namely´ fibroadenoma, invasive ductal/lobular/papillary carcinoma, hyperplasia (ductal/lobular), and lobular carcinoma *in situ*. Statistical analyses were two-tailed and set at a confidence interval of 95%.

### Ethical considerations

The study was approved by the Medical Ethics Committee of the Prince Sattam Bin Abdulaziz University (Ethics approval number REC-HSD-72-2021). Informed consent was obtained from the patients (or their legal guardians, where applicable) before participating in the study. Patient information obtained from electronic health records was completely anonymized.

## Results

### General characteristics

Data was included from a total of 270 participants who met the selection criteria. The study population was all-female, aged 33.7 ± 10.75 years, mostly Saudis, 188 (69.6%), without comorbid diabetes mellitus, 233 (86.3%), nor a family history of breast cancer, 209 (77.4%). Patients presented with an array of symptoms clinically suggestive of breast tumors. The most frequently encountered symptom was a breast lump, 259 (95.9%), followed by pain, 51 (18.9%), breast abscess, 13 (4.8%) and nipple discharge, 7 (2.6%). Most were negative for cancer as per breast mammogram, FNAC or breast lump biopsy, or lymph node biopsies, 262 (97%), 233 (86.3%), and 239 (88.5%), respectively. Nearly all women suffered ailments in either breast, 262 (97%), while a small few had bilateral afflictions, 8 (3%). Most underwent a standard excision biopsy, 245 (90.7%). A tabulation of demographic characteristics, medical history, and diagnostic investigations is shown in Table 1.

**Table 1:** demographic characteristics, medical history and diagnosis of the patients

Demographic characteristics of patients		Frequency	Percentages
Age (In years)	30 years or less	110	40.7%
	31-45 years	114	42.3%
	46 years or more	46	17%
	Mean± SD(Range)	33.7 ± 10.75 (17-79) years	
Residence	Non-Saudi	82	30.4%
	Saudi	188	69.6%
Family history of Breast cancer	Yes	61	22.6%
	No	209	77.4%
Diabetes Mellitus	Yes	37	13.7%
	No	233	86.3%
Breast mammogram	Positive	8	3%
	Negative	262	97%
FNA/Biopsy	Positive	37	13.7%
	Negative	233	86.3%
Surgery type	Excision	245	90.7%
	Incision	5	1.9%
	Radical mastectomy	5	1.9%
	N/A	15	5.6%
LNB biopsy	Negative	239	88.5%
	Positive	26	9.6%
	N/A	5	1.9%
Sides	Both	8	3%
	Left	104	38.5%
	Right	158	58.5%
Symptoms	Breast lump	259	95.9%
	Mastodynia	35	13%
	Pain	22	8.1%
	Breast abscess	13	4.8%
	Breast Discharge	7	2.6%
	Dizziness	4	1.5%
	Giddiness	4	1.5%
	Hypertrophy	3	1.1%
	Gastritis	2	0.7%
	Nausea vomiting	2	0.7%
	Discomfort	2	0.7%

### Histopathological findings

The distribution of histopathological diagnosis among our study population is tabulated in Table 2. More than half the population, 143 (53%), had a histopathological diagnosis of fibroadenoma. Invasive ductal/lobular/papillary carcinoma and hyperplasia (ductal/lobule) were the other two frequently encountered histological diagnoses, 43 (15.9%) and 18 (6.7%), respectively.

### Tumor association and associated factors

Those patient characteristics that were significant on bivariate analysis (p < 0.25) were used as independent covariates to model binary logistic regressions predicting the likelihood of the three most frequently encountered histological tumor diagnoses viz. Fibroadenoma, invasive ductal/lobular/papillary carcinoma, and hyperplasia (ductal/lobular). These associations are depicted in Table 3, Table 4 and Table 5.

**Table 2:** distribution of histopathological diagnosis

Histopathological diagnosis	Frequency	Percentage
Fibroadenoma	143	53%
Invasive ductal/lobular/papillary carcinoma	43	15.9%
Hyperplasia (ductal/lobule)	18	6.7%
Lobular CIS	18	6.7%
Medullary carcinoma	15	5.6%
Phyllodes tumor	14	5.2%
Fat necrosis	13	4.8%
Breast papilloma	13	4.8%
Mastitis	11	4.1%
Tubular adenoma	10	3.7%
Lipomatous neoplasm	8	3%
Sclerosis Adenosis	7	2.6%
Intraductal papilloma/papillary	6	2.2%
Ductal CIS	5	1.9%
Malignant neoplasm	5	1.9%
Papillary CA	2	0.7%

**Table 3:** association of “fibroadenoma” with associated factors

Demographic characteristics of patients		Fibroadenoma		Unadjusted OR(95% CI; Sig)	Adjusted OR(95% CI)
		Yes	No		
Age (In years)	≤30 years	76(69.1%)	34(30.9%)	Reference(1)	Reference(1)
	31-45 years	61(53.5%)	53(46.5%)	14.9(5.7-38.5; <0.001)	0.29(0.15-0.58; <0.001)
	>46 years	6(13%)	40(87%)	7.67(3-19.52; <0.001)	0.05(0.02-0.16; <0.001)
Residence	Non-Saudi	36(43.9%)	46(56.1%)	Reference(1)	Reference(1)
	Saudi	107(56.9%)	81(43.1%)	1.69(1-2.8; 0.05)	1.21(0.62-2.38; 0.57)
Family history of Breast cancer	Yes	23(37.7%)	38(62.3%)	0.45(0.25-0.81; 0.007)	0.34(0.15-0.77; 0.010)
	No	120(57.4%)	89(42.6%)	Reference(1)	Reference(1)
Diabetes mellitus	Yes	16(43.2%)	21(56.8%)	0.64(0.32-1.28; 0.205)	0.64(0.32-1.28; 0.205)
	No	127(54.5%)	106(45.5%)	Reference(1)	Reference(1)
Sides	Both	6(75%)	2(25%)	Reference(1)	Reference(1)
	Left	65(62.5%)	39(37.5%)	3.58(0.70-18.3; 0.125)	0.16(0.02-1.08; 0.06)
	Right	72(45.6%)	86(54.4%)	1.99(1.2-3.338.5; 0.008)	0.09(0.01-0.62; 0.014)
Breast lump	Yes	143(55.2%)	116(44.8%)	28.3(1.65-485.9; 0.021)	N/A
	No	0(0%)	11(100%)	Reference(1)	---
Mastodynia	Yes	5 (14.3%)	30(85.7%)	0.12(0.04-0.31; <0.001)	0.07(0.02-0.28; <0.001)
	No	138(58.7%)	97(41.3%)	Reference(1)	Reference(1)
Pain	Yes	17 (77.3%)	5(22.7%)	3.29(1.18-9.2; 0.023)	4.78(1.41-16.39.2; 0.012)
	No	126(50.7%)	122(49.2%)	Reference(1)	Reference(1)

Binary logistic regression used, CI= Confidence interval; Univariate analysis=Unadjusted Odds ratio (OR), Significance level <0.25; Multivariate analysis= Adjusted Odds ratio (OR), Significance level<0.05.

**Table 4:** association of “invasive ductal/lobular/papillary carcinoma” with associated factors

Demographic characteristics of patients		Invasive ductal / lobular/ papillary carcinoma		Unadjusted OR(95% CI; Sig)	Adjusted OR(95% CI)
		Yes	No		
Age (In years)	≤30 years	19(17.3%)	91(82.7%)	Reference(1)	Reference(1)
	31-45 years	12(10.5%)	102(89.5%)	0.56(0.56-1.22; 0.147)	0.46(0.21-1.04; 0.062)
	>46 years	12(26.1%)	34(73.9%)	1.69(0.74-3.85; 0.211)	0.92(0.37-2.47; 0.925)
Residence	Non-Saudi	11(13.4%)	71(86.6%)	Reference(1)	---
	Saudi	32(17%)	156(83%)	1.32(0.63-2.78; 0.457)	---
Family history of Breast cancer	Yes	15(24.6%)	46(75.4%)	2.11(1.04 -4.27; 0.038)	1.87 (0.88-0.3.98; 0.104)
	No	28(13.4%)	181(86.6%)	Reference(1)	Reference(1)
Diabetes Mellitus	Yes	11(29.7%)	26(70.3%)	2.66(1.19-5.9; 0.016)	2.43(0.99-5.95; 0.052)
	No	32(13.7%)	201 (86.3%)	Reference(1)	Reference(1)
Sides	Both	2(25%)	6(75%)	Reference(1)	---
	Left	17(16.3%)	87(83.7%)	0.58(0.11-3.15; 0.534)	---
	Right	24(15.2%)	134(84.8%)	0.54(0.10-2.82; 0.537)	---
Breast lump	Yes	42(16.2%)	217(83.8%)	1.94(0.24-15.5; 0.534)	---
	No	1(9.1%)	10(90.9%)	Reference(1)	---
Mastodynia	Yes	8(22.9%)	27(77.1%)	1.69(0.71-4.03; 0.234)	1.79(0.73-4.39; 0.205)
	No	35(14.9%)	200(85.1%)	Reference(1)	Reference(1)
Pain	Yes	4 (18.2%)	18(81.8%)	1.19(0.38-3.71; 0.763)	---
	No	39(15.7%)	209(84.3%)	Reference(1)	---

Binary logistic regression used, CI= Confidence interval; Univariate analysis=Unadjusted Odds ratio (OR), Significance level <0.25; Multivariate analysis= Adjusted Odds ratio (OR), Significance level<0.05

**Table 5:** association of “hyperplasia (ductal/lobule)” with associated factors

Demographic characteristics of patients		Hyperplasia (Ductal/lobule)		Unadjusted OR(95% CI; Sig)	Adjusted OR(95% CI)
		Yes	No		
Age (In years)	≤30 years	3(2.7%)	107(97.3%)	Reference(1)	Reference(1)
	31-45 years	9(7.9%)	105(92.1%)	3.06(0.81-11.61; 0.101)	2.53(0.64-10.1; 0.187)
	>46 years	6(13%)	40(87%)	5.35(1.28-22.42; 0.022)	2.89(0.61-13.74; 0.183)
Residence	Non-Saudi	9(11%)	73(89%)	Reference(1)	Reference(1)
	Saudi	9(4.8%)	179(85.2%)	0.41(0.15-1.07; 0.068)	0.55(0.20-1.52; 0.248)
Family history of Breast cancer	Yes	9(14.8%)	52(85.2%)	3.85(1.45-10.18; 0.007)	3.02(1.05-8.66; 0.040)
	No	9(4.3%)	200(95.7%)	Reference(1)	Reference(1)
Diabetes Mellitus	Yes	5(13.5%)	32(86.5%)	2.64(0.88-7.91; 0.082)	1.60(0.48-5.26; 0.435)
	No	13(5.6%)	220 (94.4%)	Reference(1)	Reference(1)
Sides	Both	0(0%)	8(100%)	N./A	---
	Left	3(2.9%)	101(97.1%)	N./A	---
	Right	15(9.5%)	143(90.5%)	N./A	---
Breast lump	Yes	18(6.9%)	241(93.1%)	N./A	---
	No	0(0%)	11(100%)	N./A	---
Mastodynia	Yes	5(14.3%)	30(85.7%)	2.85(0.95-8.55; 0.062)	2.57(0.79-8.30; 0.114)
	No	13(5.5%)	222(84.5%)	Reference(1)	Reference(1)
Pain	Yes	0 (0%)	22(100%)	0(0----; 0.998)	---
	No	18(7.3%)	230(92.7%)	Reference(1)	---

Binary logistic regression used, CI= Confidence interval; Univariate analysis=Unadjusted Odds ratio (OR), Significance level <0.25; Multivariate analysis= Adjusted Odds ratio (OR), Significance level<0.05.

***Fibroadenoma:*** bivariate analysis revealed numerous associations. Age, residence, familial history of breast cancer, presence of comorbid diabetes mellitus, whether ailment was unilateral/bilateral, presence of a breast lump, and mastodynia were significantly associated with the presence of fibroadenoma, p < 0.25. In the multivariate model, age remained statistically significant in predicting a histopathological diagnosis of fibroadenoma. Those in the 31-45 years age group were 54% less likely to have fibroadenoma than those less than 30 years of age, adjusted odd´s ratio, AOR: 0.46 (0.25-0.84), p < 0.012. Those greater than 46 years of age were 95.1% less likely to present with fibroadenoma, AOR: 0.049 (0.02 - 0.15), p < 0.001, evidently depicted as 40(87%) of those aged 46 and were not diagnosed with fibroadenoma.

***Invasive ductal/lobular/papillary carcinoma:*** we found age, familial history of breast cancer, comorbid diabetes mellitus, and mastodynia to be significantly associated with invasive ductal/lobular/papillary carcinoma on bivariate analysis, p < 0.25. Apart from age, no other covariate included in the model proved significant in the multivariate regression model. Those in the 31 - 45 years age group had a 58% reduction in the likelihood of having these invasive breast carcinomas when compared to the younger, < 30 years age group, AOR: 0.42(0.18 - 0.97), a statistically significant finding, p = 0.04.

**Hyperplasia (ductal/lobular):** we found age, residence, familial history of breast cancer, and comorbid diabetes mellitus to be significantly associated with ductal or lobular hyperplasia on bivariate analysis, p < 0.25. However, on multivariate analysis, we found no significant associations.

## Discussion

This study aimed to determine demographics, clinical presentations, and their association with histopathological types of breast tumors among women from KSA. Breast lump, breast pain, breast abscess and nipple discharge were the most frequently encountered symptoms. Fibroadenoma was the most common histopathological diagnosis. Multivariate analysis revealed that only age had a significant impact on the type of breast tumors, especially on fibroadenoma and invasive ductal/lobular carcinoma.

Koo *et al*. [19] conducted a national audit of cancer diagnosis to determine typical and atypical presenting symptoms of breast cancer including 2316 women with breast cancer. They reported breast lump (83%), nipple abnormalities (7%) and breast pain (6%) as the most common presenting symptoms in the patients with breast cancer. They also reported that 1 in 6 women presents without breast lump but with a variety of symptoms seeking medical advice. These results are comparable with the present study, however, the present study reported breast lump at presentation in more that 95% cases. This difference may be due the reason that in the study by Koo *et al*. [19], all the patients were histopathologically confirmed cases of breast cancer while in the preset study the most common diagnosis was fibroadenoma - a benign breast tumor. In KSA, Al Shamlan *et al*. [20] conducted a study to determine the characteristics of breast mass detectable on breast ultrasound, reporting breast lump (63.64%) and breast pain (38.54%) as the most frequent symptoms.

As reported by the present study, Stachs *et al*. [7] have reported fibroadenoma as the most common benign breast tumor. In bivariate analysis, factor like age, residence in KSA, familial history of breast cancer, presence of comorbid diabetes mellitus, presence of a breast lump, and mastodynia showed significant associated with fibroadenoma. However, multivariate analysis revealed only the significant association of age with fibroadenoma. Fibroadenoma is the most common breast tumor in women below 30 reported in the present study as well as in previous literature [21]. A 2014 study among Saudi patients with biopsy proven benign breast disease estimated fibroadenoma to be the most common lesion, accounting for nearly 45% of all cases [22].

The present study has revealed significant association of increasing age, familial history of breast cancer, comorbid diabetes mellitus, and mastodynia with invasive ductal, lobular, or papillary carcinoma on bivariate analysis. However, multivariate analysis revealed only significant association of age with invasive ductal, lobular, or papillary carcinoma. Although not significant, the present study reveals that the risk of breast cancer increases with age in Saudi Arabia. Similarly, the previous studies have revealed that increasing age above 40 increases the risk of breast cancer [23].

This study is limited by its retrospective design, which may result in biased outcome. However, the study has some strengths. Indeed, the study has thoroughly gone through the clinical presentations of the female patients who visited the hospital for the evaluation of breast tumors. In addition, the consideration of histopathological diagnosis of the patients verifies the diagnosis.

## Conclusion

Age is an important indicator of type of breast tumors. Age below 40 is associated with fibroadenoma and increasing age above 40 is associated with breast cancer among Saudi women.

### What is known about this topic

- Breast cancer is one of the most common and leading cause of mortality among women worldwide;
- Globally, new cases of women breast cancer were reported to be 2.1 million in the year 2018;
- Breast cancer accounts for 29% of all cancers reported in Saudi Arabia.

### What this study adds

- Among patients with symptoms clinically suggestive of breast tumors, breast lump was the most frequently encountered (95.9%);
- Fibroadenoma was the most frequently encountered histological diagnosis among patients with breast disease (53%);
- Age greater than 30, patients with a positive family history for breast cancer, patients with symptoms in the right breast (as opposed to both sides), and those with mastodynia were significantly less likely to have fibroadenoma. Patients with symptoms of pain, however, were significantly more likely to have fibroadenoma.
